# Diagnostic Accuracy of Rectus Femoris Ultrasound for Identifying Sarcopenia as Defined by the European Working Group on Sarcopenia in Older People 2 in Haemodialysis Patients

**DOI:** 10.3390/diagnostics16162490

**Published:** 2026-08-07

**Authors:** Judith de la Torre, Anna Arnau., Alba Singla, Miren Iriarte, Josep Mª Galceran, Joan Manel Díaz, Vicencs Esteve-Simó

**Affiliations:** 1 Nephrology Department, Althaia Xarxa Assistencial Universitària de Manresa, 08243 Manresa, Spain; miriarte@althaia.cat (M.I.); jgalceran@althaia.cat (J.M.G.); 2 Doctoral Program in Medicine and Biomedical Sciences, Universitat de Vic-Universitat Central de Catalunya (UVIC-UCC), 08500 Vic, Spain; aarnau@althaia.cat; 3 Institut de Recerca i Innovació en Ciències de la Vida i de la Salut a la Catalunya Central (IRIS-CC), 08500 Vic, Spain; asingla@althaia.cat; 4 Research, Epidemiology and Biostatistics Unit, Althaia Xarxa Assistencial Universitària de Manresa, 08243 Manresa, Spain; 5 Faculty of Medicine, University of Vic-Central University of Catalonia (UVIC-UCC), 08243 Manresa, Spain; 6 Central Catalonia Chronicity Research Group (C3RG), Institut de Recerca i Innovació en Ciències de la Vida i de la Salut a la Catalunya Central (IRIS-CC), 08243 Manresa, Spain; 7 Nephrology Department, Fundació Puigvert, 08028 Barcelona, Spain; joanmanuel.diaz@umedicina.cat; 8 Nephrology Department, Consorci Sanitari de Terrassa, 08222 Terrassa, Spain; vesteve@cst.cat

**Keywords:** sarcopenia, hemodialysis, ultrasonography, bioimpedance spectroscopy, rectus femoris

## Abstract

**Background/Objectives**: Sarcopenia is highly prevalent in patients with end-stage renal disease and is associated with adverse outcomes. We evaluated the diagnostic accuracy of rectus femoris ultrasound, compared with bioimpedance spectroscopy (BIS), for identifying sarcopenia defined by the European Working Group on Sarcopenia in Older People 2 (EWGSOP2) in haemodialysis patients, and derived overall and sex-specific ultrasound cut-off values. **Methods**: In this single-centre cross-sectional study, haemodialysis patients were assessed for sarcopenia according to EWGSOP2 criteria. Rectus femoris thickness (RFT) and cross-sectional area (CSA) were measured by ultrasound, while BIS was used to estimate body composition. Receiver operating characteristic (ROC) curves were constructed, and areas under the curve (AUC) with 95% confidence intervals were estimated to assess the ability of ultrasound and BIS-derived parameters to discriminate confirmed sarcopenia. Optimal overall and sex-specific cut-off values were determined using the Youden index. **Results**: A total of 115 patients were included. Median age was 73.0 years [IQR: 63.0–82.0], 67.0% were men. Sarcopenia was diagnosed in 50 patients (43.5%; 95%CI: 34.3–53.0). RFT and CSA showed moderate diagnostic accuracy for confirmed sarcopenia, with AUCs of 0.73 (95%CI: 0.63–0.82) and 0.72 (95%CI: 0.63–0.81), respectively. Ultrasound cut-offs of 11.9 mm for RFT and 4.4 cm^2^ for CSA yielded high sensitivity and moderate specificity. **Conclusions**: Rectus femoris ultrasound represents a feasible bedside approach for identifying EWGSOP2-defined sarcopenia in haemodialysis patients. Its diagnostic performance was comparable to that of BIS-derived measures, supporting its potential use in routine clinical practice. Further studies are warranted to validate the proposed cut-off values.

## 1. Introduction

Since 2016, sarcopenia has been recognized as a muscle disease and assigned an ICD-10-CM diagnosis code (M62.84) [1]. The revised European Working Group on Sarcopenia in Older People (EWGSOP2) definition identifies low muscle strength as the primary indicator of sarcopenia, emphasizing its role over low muscle mass in the diagnostic process [2]. Sarcopenia is associated with several adverse health-related outcomes, increasing the risk of falls, disability, and mortality [3]. Consequently, it reduces the independence and quality of life of those affected [4].

Patients with chronic kidney disease (CKD) suffer from several metabolic abnormalities, such as accumulation of uremic toxins, acidosis, multiple endocrine disorders and chronic systemic inflammation driven by pro-inflammatory cytokines that, alongside the dialysis procedure, induce a loss of energy stores, reduction in protein synthesis and increase in catabolism defining the protein-energy wasting (PEW) syndrome [5,6]. Consequently, assessing muscle mass loss has become a pivotal indicator of nutritional status, as it allows for the clinical differentiation of patients suffering from this condition.

A large number of studies have reported a high prevalence of sarcopenia in patients with CKD, especially in advanced stages of the disease. A recent meta-analysis conducted by Duarte et al. [7] reported a pooled sarcopenia prevalence of 24.5% (95%CI: 20.9–28.3%) among individuals with CKD, noting that patients on dialysis showed a higher prevalence of severe sarcopenia and low muscle strength compared to those not undergoing dialysis. However, reported prevalence fluctuates widely based on the criteria used, the diagnostic approach, and the specific cut-off values. In accordance with EWGSOP2 algorithm, the standard approach for sarcopenia screening begins with de SARC-F questionnaire [8] to identify probable cases, followed by handgrip strength (HG) [9] and muscle mass assessments to confirm the diagnosis. Finally, physical performance is evaluated using the chair-rising test, gait speed, or the Short Physical Performance Battery to determine sarcopenia severity [10].

Although Computed tomography (CT) and Magnetic Resonance Imaging (MRI) remain the gold standard for body composition measurement, their clinical use is hindered by high cost, technical complexity, and limited accessibility. Furthermore, CT scans carry the significant limitation of exposing patients to ionizing radiation. Similarly, Dual-Energy X-ray Absorptiometry (DXA) is limited by its lack of portability and sensitivity to hydration fluctuations especially in hemodialysis (HD) patients [11,12]. Consequently, Bioelectrical Impedance Analysis (BIA) has emerged as a practical, non-invasive, and cost-effective bedside alternative, although it faces similar limitations regarding fluid overload interference [13]. Along similar lines, electrical impedance myography is an interesting emerging technique that may complement muscle evaluation, although its role in human sarcopenia, especially in kidney disease, remains underexplored and requires further investigation [14].

In recent years, musculoskeletal ultrasound has emerged as a promising, non-invasive, bedside tool for the assessment of muscle wasting in CKD and hemodialysis populations. Ultrasound-derived metrics such as muscle thickness, cross-sectional area, and echogenicity have been associated with skeletal muscle loss and PEW [15], supporting their potential role as practical complements to conventional body composition methods. In particular, rectus femoris ultrasound has attracted interest because of its accessibility and reproducibility.

The main aim of this study was to assess the diagnostic accuracy of rectus femoris ultrasound for detecting EWGSOP2-defined sarcopenia in haemodialysis patients and to compare its performance with that of BIS. The secondary aim was to establish overall and sex-specific ultrasound cut-off values for identifying sarcopenia.

## 2. Materials and Methods

### 2.1. Design

This was an observational, cross-sectional, single-centre study conducted between March 2024 and July 2025. The study was approved by an independent ethics committee (Fundació Unió Catalana Hospitals; CEIC 22/47) on 16 June 2022 and was carried out in accordance with the Declaration of Helsinki and all local legal and regulatory requirements. Written informed consent was obtained from all participants.

### 2.2. Settings and Subjects

We included adult patients (≥18 years old) on maintenance haemodialysis (HD) with a treatment vintage of at least three months at the HD unit of Hospital Sant Joan de Déu, Althaia Xarxa Assistencial Universitària de Manresa. Patients were receiving treatment three times per week. Exclusion criteria included limb amputation, advanced terminal illness or neuromuscular disease, prolonged hospitalization within the two months prior to the study and the presence of a pacemaker.

### 2.3. Sample Size

A total sample of 115 patients, assuming a sarcopenia prevalence of 28.5% [16], achieves 91% power to detect a difference of 0.2 between the area under the receiver operating characteristic (ROC) curve under the null hypothesis of 0.50 and an AUC under the alternative hypothesis of 0.70 [17] using a two-sided z-test at a significance level of 0.05. Continuous test results were assumed, and the AUC was calculated over the full range of false-positive rates (0–1), assuming equal standard deviations in both groups. The sample size was calculated using Power Analysis and Sample Size Software 2021 (PASS, NCSS Statistical Software, Kaysville, UT, USA) [18].

### 2.4. Data Collection and Measures

Eligible patients were identified from the haemodialysis unit census, and potential candidates were screened approximately one week before the study visit. Baseline sociodemographic and clinical data, including age, sex, smoking status, body mass index, primary renal disease, cardiovascular comorbidities, and dialysis vintage were retrieved from medical records upon study enrolment. Patients were scheduled for a study visit on their mid-week HD day, before the dialysis session. During this visit, a single trained investigator administered the SARC-F questionnaire and measured muscle strength using handgrip dynamometry. Nutritional status was evaluated by nursing staff using the Malnutrition Inflammation Score (MIS). Approximately 30 min before its completion, rectus femoris ultrasound was performed by the same investigator, and bioimpedance spectroscopy (BIS) was carried out 20–30 min after the end of the session by nursing assistants.

### 2.5. Laboratory Tests

Blood samples were obtained from the arteriovenous fistula immediately before the mid-week haemodialysis session, on the same day as the other study measurements. To evaluate nutritional and metabolic status, albumin, prealbumin and pre-dialysis creatinine were measured using an automated analyser. C-reactive protein (CRP) and interleukin-6 (IL-6) were determined as markers of systemic inflammation. Dialysis adequacy, expressed as Kt/V, was obtained from the dialysis monitor as the mean value over one month.

### 2.6. Muscle Strength

Muscle strength was measured using a digital handgrip (HG) dynamometer (Jamar Plus+; Performance Health Supply, Inc., Cedarburg, WI, USA). Measurements were conducted before the HD session with the patient in a seated position, the elbow flexed at 90 degrees, and the forearm in a neutral position. Two measurements were obtained on the non-fistula arm, and the highest value was recorded in kilograms.

### 2.7. Bioimpedance Measurement

Body composition was analyzed using a multifrequency bioimpedance spectroscopy (BIS) device (BCM Body Composition Monitor, Fresenius Medical Care, Bad Homburg, Germany). The device measures impedance at 50 frequencies, ranging from 5 kHz to 1 MHz. By applying the Cole model and specialized fluid management algorithms, it distinguishes between extracellular water and intracellular water. Unlike standard BIA, this spectroscopy method allows for the objective quantification of overhydration and provides normalized indices for nutritional evaluation, such as the Lean Tissue Index (LTI) and Fat Tissue Index (FTI). Appendicular skeletal muscle mass (ASM) was estimated from BCM-derived parameters using the validated prediction equation developed by Lin et al. [19]. The ASM index (ASMI) was calculated by dividing ASM by height squared (kg/m^2^). Measurements were performed 30 min after completion of the HD session to allow for fluid stabilization, following established clinical protocols for post-dialysis assessment [20].

### 2.8. Muscle Ultrasound

Ultrasound measurements were performed by a single experienced investigator to ensure consistency. With the patient in a supine position and the dominant leg fully relaxed in passive extension, the rectus femoris cross-sectional area (CSA) was assessed using B-mode ultrasonography (MyLab X5, Esaote, Genoa, Italy) with a multifrequency linear transducer operating at 4–15 MHz. The examination was performed towards the end of the dialysis session, when most of the ultrafiltration had already occurred to minimize the confounding effect of residual fluid overload. The measurement site was located at two-thirds of the distance between the anterior superior iliac spine and the superior border of the patella, following international SARCUS recommendations for quadriceps muscle ultrasound and within the conceptual framework of nutritional ultrasound^®^ described by García-Almeida et al. [21,22]. The transducer was placed perpendicular to the long axis of the thigh to obtain a transverse image, using ample coupling gel and minimal pressure to avoid muscle compression. A musculoskeletal preset was used with a penetration depth of approximately 4 cm, and gain and focus were adjusted to optimize the contrast between subcutaneous tissue and muscle without saturating deeper structures. Representative images were frozen and subsequently analysed using proprietary software; a cursor was used to trace the inner echogenic boundary (fascia) of the muscle to determine CSA and the rectus femoris thickness (RFT). All parameters represent the average of three consecutive readings. Ultrasound measurements were performed blinded to the BIS results.

### 2.9. Sarcopenia Diagnosis

Sarcopenia was diagnosed according to the 2019 criteria of the EWGSOP2. The SARC-F questionnaire was administered as a screening tool to identify individuals at risk; it was not used for diagnostic classification. Muscle strength was evaluated using handgrip strength; values below 27 kg in men and 16 kg in women were considered indicative of probable sarcopenia. Muscle mass was assessed using bioimpedance spectroscopy, and low muscle mass was defined as an ASMI < 7.0 kg/m^2^ in men and <5.5 kg/m^2^ in women. Confirmed sarcopenia was defined by the coexistence of low muscle strength and low ASMI. Physical performance tests were collected within the broader study protocol but were not included in the present analysis, as the focus of this manuscript was diagnostic accuracy for sarcopenia overall.

### 2.10. Statistical Analysis

Continuous variables were expressed as mean and standard deviation (SD) or median and interquartile range, according to their distribution. Normality was assessed visually and using the Shapiro–Wilk test. Categorical variables were summarised as absolute and relative frequencies. Between-group comparisons (sarcopenic vs. non-sarcopenic patients, and sex-specific analyses) were performed using Student’s test or Mann–Whitney U test for continuous variables and the chi-square test or Fisher’s exact test for categorical variables, as appropriate. Receiver operating characteristic (ROC) curves were constructed to evaluate the diagnostic accuracy of ultrasound-derived parameters (rectus femoris muscle thickness and cross-sectional area) and bioimpedance-derived variables (body cell mass, phase angle and related indices) for EWGSOP2-confirmed sarcopenia. The area under the ROC curve (AUC) and its 95% confidence interval were calculated, and optimal cut-off points were derived using the Youden index for the overall cohort and separately for men and women. For each cut-off, sensitivity, specificity, positive predictive value; negative predictive value, positive likelihood ratio, negative likelihood ratio and their 95% confidence intervals were estimated. All statistical tests were two-sided, and a *p* value less than 0.05 was considered statistically significant. Statistical analyses were performed using R version 4.3.1 (R Core Team, 2023) within the RStudio integrated development environment (Posit Team, 2024).

## 3. Results

**Figure 1:**
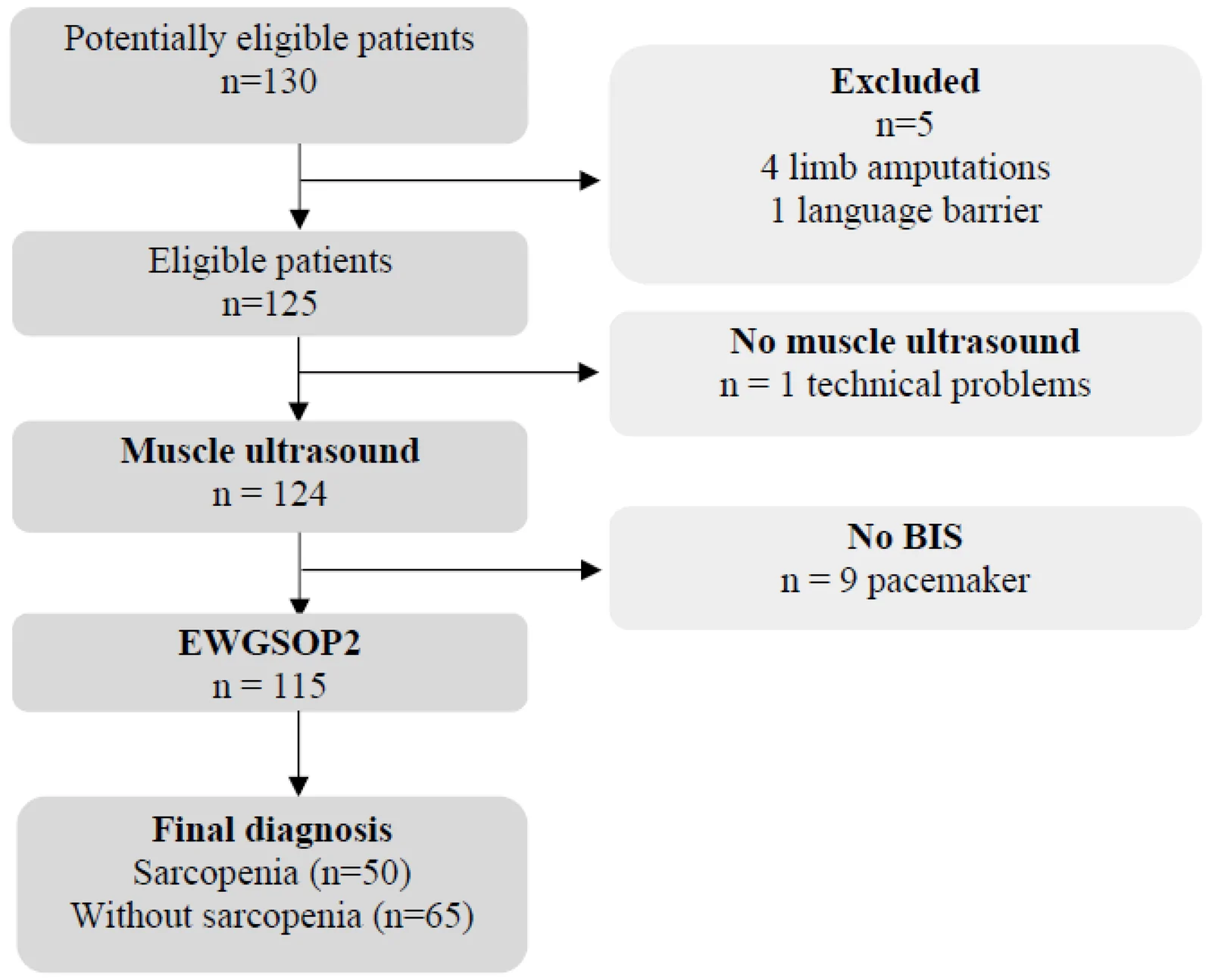
Flow of patients through the study.

A total of 130 patients were evaluated for eligibility. Of these, 14 were excluded: four due to limb amputation, one because of a language barrier and nine because the presence of a pacemaker precluded BIS measurement. One additional patient, despite meeting all inclusion criteria, was excluded from the statistical analysis due to technical difficulties during muscle ultrasound examination. Consequently, 115 patients were included in the final analysis (see Figure 1), of whom 77 were men (67.0%). The median age was 73.0 [IQR: 63.0–82.0] years, and the median duration on HD was 26.0 [IQR: 9.0–46.0] months. All other sociodemographic and clinical parameters are summarised in Table 1.

**Figure 2:**
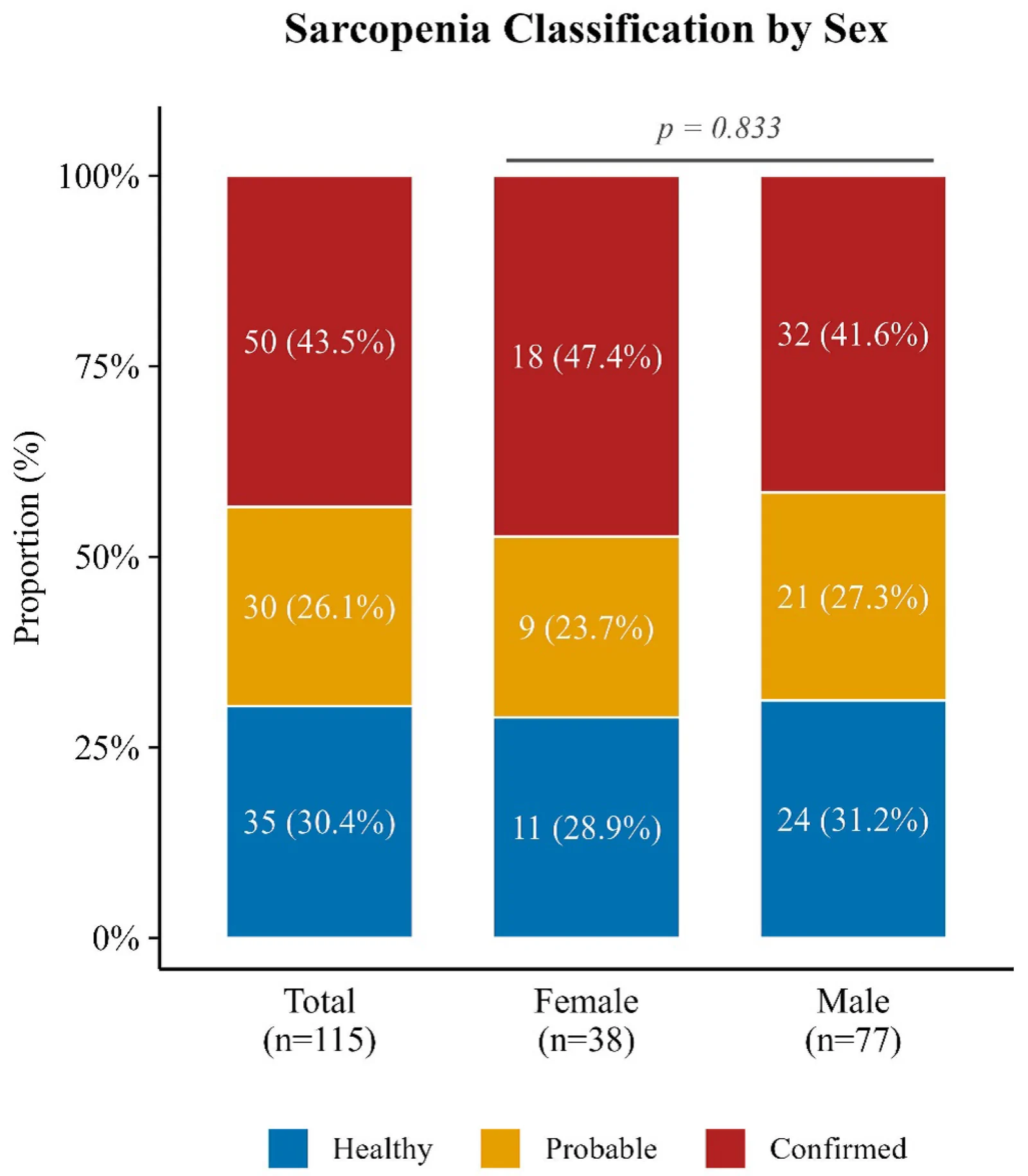
Prevalence of EWGSOP2-defined sarcopenia overall and by sex.

Sarcopenia was diagnosed in 50 out of 115 patients (43.5%; 95%CI: 34.3–53.0) according to the EWGSOP2 criteria. Of the total sample, 35 patients (30.4%) were classified as healthy, 30 (26.1%) as probable sarcopenia and 50 (43.5%) as confirmed Sarcopenia. No statistically significant differences were observed between sexes (*p* = 0.833) (see Figure 2).

Sarcopenic patients were significantly older (80.0 [IQR: 75.0–84.0] vs. 66.0 [IQR: 58.0–74.0] years; *p* < 0.001) and had lower BMI (*p* < 0.001) compared to non-sarcopenic patients. Notably, dialysis vintage did not differ significantly between groups (27.0 [9.0–50.0] vs. 26.0 [10.0–41.0] months; *p* = 0.948). All BIS-derived parameters were statistically significantly lower in the sarcopenic group, including total body water (TBW), body cell mass (BCM), phase angle (PA), LTI and FTI. Ultrasound parameters were also significantly lower: rectus femoris CSA (3.5 [IQR: 2.8–4.2] vs. 4.6 [IQR: 3.6–5.7] cm^2^; *p* < 0.001) and RFT (10.2 [IQR: 8.5–12.0] vs. 12.5 [IQR: 10.8–16.0] mm; *p* < 0.001). Full details are presented in Table 2.

ROC curve analysis was performed to evaluate the diagnostic accuracy of BIS- and ultrasound-derived parameters for sarcopenia detection. AUCs are presented in Table 3. BCM and PA were evaluated as independent BIS-derived parameters and were not included in the ASMI calculation, in order to avoid overlap with the reference standard. BCM showed the highest overall discriminative ability (AUC = 0.77 [95%CI: 0.68–0.86]; *p* < 0.001; cut-off 17.65 kg), with good performance in both males (AUC = 0.81 [95%CI: 0.72–0.91]; cut-off 18.85 kg) and females (AUC = 0.74 [95%CI: 0.58–0.91]; cut-off 15.2 kg). PA also showed good overall accuracy (AUC = 0.75 [95%CI: 0.66–0.84]; *p* < 0.001; cut-off 4.8°), with acceptable performance in males (AUC = 0.78 [95%CI: 0.68–0.88]; cut-off 4.47°), but not reaching statistical significance in females (AUC = 0.66 [95%CI: 0.47–0.84]; *p* = 0.11.

**Figure 3:**
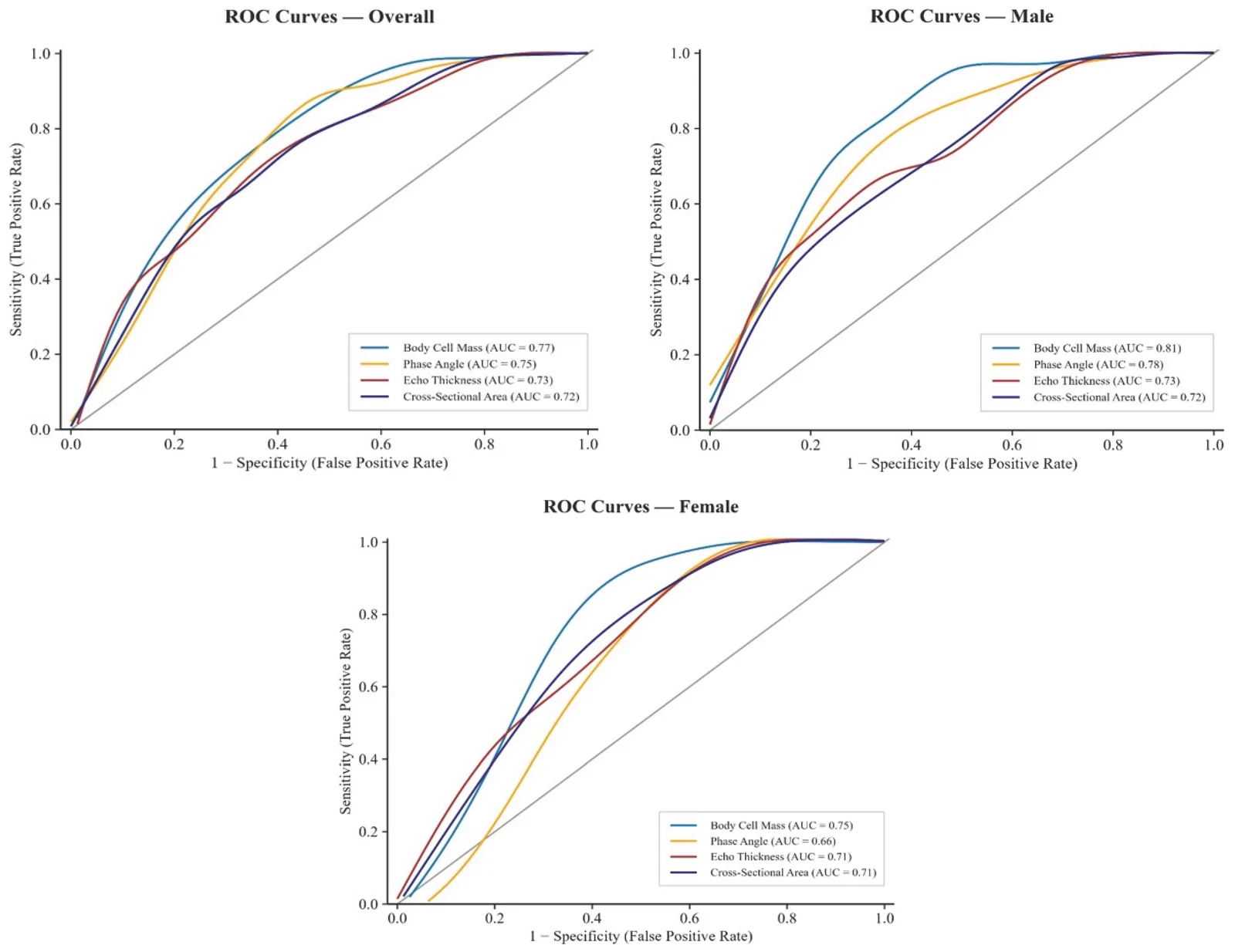
Receiver operating characteristic (ROC) curves of ultrasound-derived parameters and bioimpedance-derived variables for EWGSOP2-confirmed sarcopenia.

Regarding ultrasound parameters, RFT showed moderate overall diagnostic accuracy (AUC = 0.73 [95%CI: 0.63–0.82]; *p* < 0.001; cut-off 11.85 mm), with similar performance in males (AUC = 0.73 [95%CI: 0.62–0.85]; cut-off 11.85 mm) and females (AUC = 0.71 [95%CI: 0.54–0.88]; cut-off 11.95 mm). CSA yielded comparable results (AUC = 0.72 [95%CI: 0.63–0.81]; *p* < 0.001; cut-off 4.44 cm^2^), with consistent performance across sexes (males: AUC = 0.72 [95%CI: 0.61–0.84]; cut-off 4.44 cm^2^; females: AUC = 0.71 [95%CI: 0.55–0.88]; cut-off 3.69 cm^2^). Full results are presented in Table 3. The corresponding ROC curves are illustrated in Figure 3.

## 4. Discussion

In the present study, the prevalence of EWGSOP2-defined sarcopenia was 43.5%. This prevalence exceeds the pooled estimate of 24.5% (95% CI: 20.9–28.3%) reported by Duarte et al. (2024) [7] in a global systematic review and meta-analysis of 140 studies comprising 42,041 patients across the entire CKD spectrum; however, when restricted to HD patients diagnosed using the EWGSOP2 definition, their pooled prevalence rose to 30.6% (95% CI: 22.3–39.5%), narrowing the gap with our findings. The higher prevalence observed in our cohort may partly reflect sample-specific characteristics, including age distribution, comorbidity burden, and time on dialysis, all of which are recognised risk factors for sarcopenia in this population.

With regard to sex, sarcopenia prevalence was 41.6% in men and 47.4% in women, with no statistically significant difference (*p* = 0.554), consistent with the virtually identical rates reported by Duarte et al. in men (25.8%) and women (25.7%; *p* = 0.96). This absence of a sex-based difference contrasts with findings in the general population, where sarcopenia tends to predominate in men [23]. In the CKD setting, the uraemic environment may attenuate hormonal differences between sexes, resulting in a similarly elevated catabolic burden regardless of sex.

These findings are further supported by a recent Spanish study conducted by De la Flor et al. (2025) [24] in 74 maintenance HD patients, in which the overall prevalence of confirmed sarcopenia was 40.5%, closely mirroring our results (43.5%), and both studies share a comparable diagnostic framework conducted in Spanish hemodialysis units. In contrast to our findings, however, De la Flor et al. reported a significantly higher prevalence in men compared to women (48.1% vs. 22.7%; *p* = 0.01), a discrepancy that may partly reflect the limited number of women in both cohorts (22 in De la Flor et al. and 38 in the present study), which reduces statistical power and may hinder the detection of true sex-based differences, or conversely, produce spurious significant results in small subgroups.

In our cohort, sarcopenic patients were significantly older than non-sarcopenic patients, consistent with the well-established role of age as a primary risk factor for sarcopenia in this population, as confirmed in the meta-analysis by Zhang et al. (2025) [25], in which older age was the strongest independent predictor of sarcopenia among 2904 dialysis patients (SMD = 0.76, 95% CI: 0.54–0.99; *p* = 0.001).

Notably, dialysis vintage did not differ significantly between groups. Although this might seem counterintuitive given the chronic catabolic burden associated with prolonged dialysis exposure, this finding is consistent with evidence from large meta-analyses. Shu et al. (2022) [16], in a systematic review of 6,162 dialysis patients, reported that dialysis duration did not significantly influence sarcopenia prevalence in meta-regression analysis (regression coefficient 0.002; 95%CI: −0.002 to 0.005; *p* = 0.327), and similar results were reported by the large multicentre SARC-HD cohort (Mondini et al., 2025) [26]. Together, these findings suggest that patient-intrinsic characteristics such as age may play a more prominent role in the development of sarcopenia than the duration of dialysis itself.

Regarding nutritional status, sarcopenic patients showed lower albumin and prealbumin levels, reinforcing the close link between protein–energy malnutrition and muscle loss in HD. In addition, a higher proportion of sarcopenic patients met the MIS >7 threshold proposed by Arias-Guillén et al. in the Nutrendial study [27] to diagnose PEW, indicating that this score identifies a subgroup at increased risk of concomitant malnutrition and sarcopenia in this population. In parallel, sarcopenic patients also exhibited higher IL-6 and CRP concentrations, consistently pointing to a pro-inflammatory milieu as an additional contributor to muscle wasting in this setting. Although Kt/V values were slightly higher in sarcopenic patients, this finding should be interpreted with caution, as higher dialysis dose may reflect differences in body size rather than a direct protective or harmful effect on muscle mass. In our cohort, sarcopenic patients were also more frequently treated with conventional haemodialysis than with online haemodiafiltration, but the cross-sectional design and limited sample size do not allow any causal inferences to be drawn.

Musculoskeletal ultrasound has emerged as a promising bedside tool for sarcopenia, offering a direct regional evaluation of muscle quantity and quality and overcoming some of the hydration-related limitations of conventional BIA. The recent literature has further expanded the scope of ultrasonography in dialysis patients to include promising techniques such as shear wave elastography, which may provide additional information on muscle stiffness and tissue properties as indirect markers of systemic muscle impairment [28], although further studies are needed to clarify its role in dialysis populations. Within this broader context, quadriceps ultrasound has been increasingly explored in HD patients, and the rectus femoris has emerged as the most informative muscle among the quadriceps components and other muscle groups studied for detecting muscle wasting, both in the context of PEW and sarcopenia.

The first studies applying quadriceps ultrasound in HD focused primarily on nutritional status. Sabatino et al. [29] reported that RFT and vastus intermedius thickness (VIT) were markedly reduced in HD patients and showed significant associations with established markers of malnutrition. Building on these findings, Battaglia et al. [15] confirmed lower RFT in HDT patients compared with healthy controls and demonstrated strong correlations between ultrasound-derived quadriceps measurements and BIS parameters such as BCM and PA, further supporting the value of RF ultrasound as a bedside marker of muscle wasting in this population.

In a larger multicentre cohort, Sahathevan et al. [30] extended these observations by evaluating quadriceps ultrasound specifically in relation to PEW defined by ISRNM criteria. They showed that patients with PEW had significantly smaller RF and VI thickness and RFCSA, and that RFCSA provided better discrimination of PEW than BIS, with an AUC of 0.69 (95%CI: 0.62–0.75; *p* < 0.001). Using this approach, they proposed sex-specific CSA cut-offs of <6.00 cm^2^ in men and <4.47 cm^2^ in women, and individuals below these thresholds had an odds ratio of 8.0 for meeting ISRNM criteria for PEW. In addition, a small Japanese study by Matsuzawa et al. [31] supported the validity of rectus femoris CSA by ultrasound as a surrogate for BIA-derived low muscle mass in HD patients, but it did not directly evaluate diagnostic performance for clinically defined sarcopenia.

In an Egyptian cohort of 41 HD patients, Nagy et al. [17] were the first to focus specifically on sarcopenia diagnosed using the EWGSOP 2010 criteria, classifying patients as sarcopenic or non-sarcopenic based on low muscle mass by BIA together with low muscle strength and/or impaired physical performance. Among the different ultrasound measurements, quadriceps muscle CSA was the only significant predictor of sarcopenia, with a ROC-derived AUC of 0.74 and an optimal cut-off of 2.92 cm^2^ (sensitivity 75%, specificity 73.3%) for the whole sample, and very similar thresholds in men and women. However, this study was conducted in a relatively small, non-Caucasian cohort with a younger mean age and clinical characteristics that differ substantially from those of typical European HD populations, which may limit the direct applicability of its proposed quadriceps CSA cut-offs to our setting.

In our study, we primarily aimed to evaluate the diagnostic utility of rectus femoris ultrasound for EWGSOP2-confirmed sarcopenia in a predominantly Caucasian HD population and to derive sex-specific cut-offs that are applicable to our clinical setting. In parallel, we assessed several BIS-derived parameters to identify which body composition measures best complement ultrasound in the diagnostic work-up of sarcopenia. In this cohort, RFT and CSA showed moderate diagnostic accuracy for EWGSOP2-confirmed sarcopenia (AUCs 0.73 and 0.72, respectively), with high sensitivity and modest specificity at optimal Youden cut-offs (RFT 11.9 mm; RFCSA 4.4 cm^2^). Ultrasound performance was comparable to that of PhA (AUC 0.75), supporting its role as a practical tool for detecting sarcopenia in routine HD care. Accordingly, sex-specific thresholds of approximately 12 mm in men and women for RFT and 4.4 cm^2^ in men and 3.7 cm^2^ in women for CSA, emerged as the most appropriate cut-offs for identifying sarcopenia in this population. Although not the primary focus of our study, BCM achieved the highest overall discriminative ability for confirmed sarcopenia (AUC 0.77), with a Youden-derived cut-off of 17.7 kg providing a reasonable balance between sensitivity and specificity.

Interestingly, when compared with the recent Spanish cohort reported by De la Flor et al. [24], which had a similar mean age and prevalence of EWGSOP2-confirmed sarcopenia, our sex-specific rectus femoris cut-offs were clearly higher (around 12 mm and 4.4/3.7 cm^2^ vs. their proposed thresholds of approximately 8 mm and 2.4 cm^2^). These discrepancies may reflect differences in ultrasound protocols, inter-operator variability or patient case-mix between studies, as well as the relatively small sample sizes, and highlight the challenge of defining universal quadriceps-based cut-offs even across apparently similar HD populations. In addition, the DRECO study [32], carried out in hospitalised patients at nutritional risk, reported rectus femoris CSA and RFT cut-offs of 3.66 cm^2^ and 9.66 mm, respectively, for confirmed sarcopenia, which are numerically close to, but still slightly lower than, the thresholds identified in our HD cohort.

The present study has several limitations that should be acknowledged. First, it was conducted in a single centre with a relatively small sample size, which may limit the generalisability of our findings to other HD populations and healthcare systems. The female subgroup was also relatively small; therefore, sex-specific cut-offs should be interpreted as exploratory and not as definitive clinical thresholds. In addition, the cross-sectional design does not allow us to infer causality or to evaluate the prognostic value of the proposed cut-offs for hard outcomes such as hospitalisation or mortality. We also excluded patients with limb amputation or with very limited mobility due to advanced degenerative or terminal disease, which may have introduced selection bias by under-representing the most severely disabled individuals. The timing of ultrasound towards the end of the dialysis session and BIS after dialysis was chosen to reduce fluid-related confounding; however, residual acute changes in hydration status may still have affected the absolute values of both techniques. Finally, ultrasound was performed by a single trained investigator following a standardized protocol, which likely minimized operator-dependent variability. However, intraobserver reliability analysis was not performed, and formal reproducibility data were not available.

## 5. Conclusions

In this study, rectus femoris ultrasound appeared to be a useful tool to support the diagnosis of EWGSOP2-defined sarcopenia in HD patients, either as a complement to BIS or as an alternative in patients in whom bioimpedance is not available or is unreliable. Given the high prevalence of sarcopenia in end-stage renal disease and its well-documented impact on quality of life and clinical outcomes, a simple, portable and increasingly accessible technique such as rectus femoris ultrasound represents a promising and practical option for muscle assessment in dialysis units. Nevertheless, our findings, together with those of other studies, also highlight the major challenge of defining rectus femoris cut-offs that are both valid and generalisable, and underscore the need for larger multicentre, studies to externally validate and standardise these thresholds across different clinical settings. Future longitudinal studies using ultrasound would be helpful to better understand the evolution of muscle changes and their clinical significance over time in hemodialysis patients.

## Figures and Tables

**Table 1:** Baseline demographic, clinical, laboratory, body composition, and sarcopenia-related characteristics of haemodialysis patients, overall and by sex.

	All Patients	Female	Male	*p* Value
	(n = 115)	(n = 38)	(n = 77)	
Sociodemographic and clinical data:				
Age (years)	73.0 [63.0–82.0]	74.5 [63.0–83.0]	71.0 [63.0–80.0]	0.332 ^2^
Gender				
Male	77 (67.0%)			
Female	38 (33.0%)			
Race				0.100 ^2^
Caucasian	101 (93.5%)	32 (94.1%)	69 (93.2%)	
Black	3 (2.8%)	1 (2.9%)	2 (2.7%)	
Hispanic	4 (3.7%)	1 (2.9%)	3 (4.1%)	
Weight (kg)	71 [61.5–85.0]	60.0 [55.0–74.0]	75.0 [66.0–88.0]	<0.001 ^2^
Height (cm)	164.4 ± 8.4	155.9 ± 5.8	168.6 ± 6.0	<0.001 ^1^
BMI	26.3 [23.1–30.0]	26.0 [21.7–29.3]	26.8 [23.5–30.1]	0.352 ^2^
Smoking				<0.001 ^3^
Non-smoker	48 (41.7%)	33 (86.8%)	15 (19.5%)	
Smoker	12 (10.4%)	1 (2.6%)	11 (14.3%)	
Ex-smoker	55 (47.8%)	4 (10.5%)	51 (66.2%)	
Alcohol consumption				0.024 ^3^
Abstinent	84 (73.7%)	34 (89.5%)	50 (65.8%)	
Mild	22 (19.3%)	3 (7.9%)	19 (25.0%)	
Moderate	8 (7.0%)	1 (2.6%)	7 (9.2%)	
Heavy	0 (0.0%)	0 (0.0%)	0 (0.0%)	
Dialysis and clinical parameters:				
Haemodialysis duration (months)	26.0 [9.0–46.0]	33.5 [14.0–62.0]	24.0 [8.0–40.0]	0.072 ^2^
Haemodialysis hours per day	4.0 [3.8–4.2]	4.0 [3.5–4.1]	4.0 [3.8–4.2]	0.779 ^2^
Modality				0.670 ^3^
HD conventional	40 (35.1%)	14 (37.8%)	26 (33.8%)	
Online HF	74 (64.9%)	23 (62.2%)	51 (66.2%)	
KTv (mean of last month)	1.5 [1.3–1.7]	1.8 [1.6–2]	1.5 [1.3–1.6]	<0.001 ^2^
Malnutrition Inflammation Score (MIS)	6.0 [4.0–7.0]	6.5 [4.0–7.0]	5.0 [3.0–7.0]	0.114 ^2^
Well-nourished (0–7)	85 (76.6%)	29 (76.3%)	56 (76.7%)	0.963 ^3^
Malnourished (>7)	26 (23.4%)	9 (23.7%)	17 (23.3%)	
Associated Comorbidities:				
Obesity	41 (35.7%)	14 (36.8%)	27 (35.1%)	0.100 ^3^
Type 1 Diabetes	2 (1.7%)	0 (0.0%)	2 (2.6%)	0.100 ^4^
Type 2 Diabetes	53 (46.1%)	18 (47.4%)	35 (45.5%)	0.846 ^3^
Hypertension	102 (88.7%)	36 (94.7%)	66 (85.7%)	0.215 ^3^
Dyslipidaemia	74 (64.3%)	21 (55.3%)	53 (68.8%)	0.153 ^3^
Ischemic heart disease	18 (15.7%)	3 (7.9%)	15 (19.5%)	0.108 ^3^
Cerebrovascular disease	12 (10.4%)	2 (5.3%)	10 (13.0%)	0.332 ^3^
Peripheral vascular disease	15 (13.0%)	1 (2.6%)	14 (18.2%)	0.019 ^3^
Hepatic steatosis	4 (3.5%)	0 (0.0%)	4 (5.2%)	0.300 ^4^
Chronic heart failure	11 (9.6%)	6 (15.8%)	5 (6.5%)	0.174 ^3^
Etiology of chronic kidney disease:				0.449 ^3^
Nephroangiosclerosis	18 (15.8%)	4 (10.5%)	14 (18.4%)	
Diabetic Nephropathy	31 (27.2%)	12 (31.6%)	19 (25.0%)	
Interstitial Nephropathy	9 (7.9%)	3 (7.9%)	6 (7.9%)	
Glomerulonephritis	15 (13.2%)	5 (13.2%)	10 (13.2%)	
Genetic diseases	7 (6.1%)	5 (13.2%)	2 (2.6%)	
Nephrotoxins	3 (2.6%)	1 (2.6%)	2 (2.6%)	
Others	13 (11.4%)	4 (10.5%)	9 (11.8%)	
Unknown	18 (15.8%)	4 (10.5%)	14 (18.4%)	
Laboratory data:				
Albumin (g/dL)	4 ± 0.3	4 ± 0.3	4 ± 0.3	0.450 ^1^
Prealbumin (mg/dL)	24.2 [19.9–28.8]	24.2 [19.5–29.5]	24.3 [21–27.6]	0.950 ^2^
Creatinine (mg/dL)	6.6 [5.6–8.2]	6.2 [5.2–7.2]	6.9 [5.9–8.5]	0.027 ^2^
IL-6 (pg/mL)	8.9 [5.9–16.4]	8.9 [6.2–13.3]	8.7 [5.9–16.6]	0.929 ^2^
CRP (mg/L)	4.7 [1.9–14.3]	3.1 [1.8–11]	5.8 [2–15.2]	0.301 ^2^
Sarcopenia Assessment Evaluations:				
SARC-F Survey				0.147 ^3^
Normal (<4)	77 (67%)	22 (57.9%)	55 (71.4%)	
Abnormal (≥4)	38 (33%)	16 (42.1%)	22 (28.6%)	
Handgrip Dynamometer (kg)	19.8 [13.1–25.9]	13.6 [10.1–16.6]	23 [17.9–29.4]	<0.001 ^2^
Normal (≥27 kg Male; ≥16 kg Female)	35 (30.4%)	11 (28.9%)	24 (31.2%)	0.808 ^3^
Abnormal (<27 kg Male; <16 kg Female)	80 (69.6%)	27 (71.1%)	53 (68.8%)	
BIS				
ASMI (kg/m^2^)	6.7 ± 1.4	5.7 ± 1.4	7.1 ± 1.2	<0.001 ^1^
Normal (≥7 kg Male; ≥5.5 kg Female)	56 (48.7%)	16 (42.1%)	40 (51.9%)	0.321 ^3^
Abnormal (<7 kg Male; <5.5 kg Female)	59 (51.3%)	22 (57.9%)	37 (48.1%)	
TBW (L)	33.3 ± 7.3	27.4 ± 5	36.2 ± 6.4	<0.001 ^1^
BCM	16.9 [13.3–22]	12.1 [9.5–15.8]	19.4 [15.6–24.7]	<0.001 ^2^
PhA (degrees)	4.4 [3.8–5.5]	4.3 [3.7–4.7]	4.6 [3.9–5.9]	0.034 ^2^
LTI (kg/m^2^)	12.3 ± 3.2	10.4 ± 2.8	13.2 ± 3	<0.001 ^1^
FTI (kg/m^2^)	14.4 [11.2–19.1]	17.2 [11.1–20.8]	14 [11.3–17.8]	0.084
Ultrasound (US)				
CSA (cm^2^)	3.9 [3.1–5]	3.3 [2.5–4.2]	4.3 [3.6–5.4]	<0.001 ^2^
RFT (mm)	11.8 [9.7–14]	10.8 [9.3–12.5]	12.2 [10.1–14.9]	0.012 ^2^

Data expressed as mean ± SD, median [p25–p75], or number (%). ^1^ T-Test Student, ^2^ Mann–Whitney U test; ^3^ Pearson’s Chi-squared test; ^4^ Fisher’s exact test. Abbreviations: BMI: Body mass index; MIS: Malnutrition Inflammation Score; BIS: Bioelectrical Impedance Spectroscopy; ASMI: Appendicular Skeletal Muscle Index; TBW: Total body water; BCM: Body Cell Mass; PhA: Phase Angle: LTI: Lean Tissue Index; FTI: Fat Tissue Index; CSA: Cross-Sectional Area; RFT: Rectus Femoris Muscle Thickness.

**Table 2:** Baseline demographic, clinical, laboratory, body composition, and sarcopenia-related characteristics between patients with and without sarcopenia.

	All Patients	Patients Without Sarcopenia	Patients With Sarcopenia	*p* Value
	(n = 115)	(n = 65)	(n = 50)	
Sociodemographic and clinical data:				
Age (years)	73.0 [63.0–82.0]	66.0 [58.0–74.0]	80.0 [75.0–84.0]	<0.001 ^2^
Gender				0.554 ^3^
Male	77 (67.0%)	45 (69.2%)	32 (64.0%)	
Female	38 (33.0%)	20 (30.8%)	18 (36.0%)	
Race				0.100 ^4^
Caucasian	101 (93.5%)	56 (93.3%)	45 (93.8%)	
Black	3 (2.8%)	2 (3.3%)	1 (2.1%)	
Hispanic	4 (3.7%)	2 (3.3%)	2 (4.2%)	
Weight (kg)	71.0 [61.5–85.0]	83.0 [70.5–94.0]	62.8 [57.5–68.8]	<0.001 ^2^
Height (cm)	164.4 ± 8.4	166.2 ± 8.2	162.0 ± 8.3	0.012 ^1^
BMI	26.3 [23.1–30.0]	29.3 [26.2–33.0]	23.9 [21.8–25.4]	<0.001 ^2^
Smoking				0.370 ^4^
Non-smoker	48 (41.7%)	27 (41.5%)	21 (42.0%)	
Smoker	12 (10.4%)	9 (13.8%)	3 (6.0%)	
Ex-smoker	55 (47.8%)	29 (44.6%)	26 (52.0%)	
Alcohol consumption				0.260 ^4^
Abstinent	84 (73.7%)	51 (78.5%)	33 (67.3%)	
Mild	22 (19.3%)	9 (13.8%)	13 (26.5%)	
Moderate	8 (7%)	5 (7.7%)	3 (6.1%)	
Heavy	0 (0.0%)	0 (0.0%)	0 (0.0%)	
Dialysis and clinical parameters:				
Haemodialysis duration (months)	26.0 [9.0–46.0]	26.0 [10.0–41.0]	27.0 [9.0–50.0]	0.948 ^2^
Haemodialysis hours per day	4.0 [3.8–4.2]	4.0 [4.0–4.3]	4.0 [3.5–4.0]	0.012 ^2^
Modality				0.003 ^4^
HD conventional	40 (35.1%)	15 (23.4%)	25 (50.0%)	
Online HF	74 (64.9%)	49 (76.6%)	25 (50.0%)	
KTv (mean of last month)	1.5 [1.3–1.7]	1.5 [1.3–1.6]	1.6 [1.4–1.9]	0.013 ^2^
MIS	6.0 [4.0–7.0]	5.0 [3.0–6.0]	7.0 [5.0–10.0]	<0.001 ^2^
Well-nourished (0-7)	85 (76.6%)	57 (93.4%)	28 (56.0%)	<0.001 ^4^
Malnourished (>7)	26 (23.4%)	4 (6.6%)	22 (44.0%)	
Associated Comorbidities:				
Obesity	41 (35.7%)	36 (55.4%)	5 (10.0%)	<0.001 ^3^
Type 1 Diabetes	2 (1.7%)	2 (3.1%)	0 (0.0%)	0.504 ^4^
Type 2 Diabetes	53 (46.1%)	32 (49.2%)	21 (42.0%)	0.441 ^3^
Hypertension	102 (88.7%)	58 (89.2%)	44 (88.0%)	0.836 ^3^
Dyslipidaemia	74 (64.3%)	41 (63.1%)	33 (66.0%)	0.746 ^3^
Ischemic heart disease	18 (15.7%)	12 (18.5%)	6 (12.0%)	0.344 ^3^
Cerebrovascular disease	12 (10.4%)	8 (12.3%)	4 (8.0%)	0.454^4^
Peripheral vascular disease	15 (13%)	7 (10.8%)	8 (16.0%)	0.409 ^3^
Hepatic steatosis	4 (3.5%)	3 (4.6%)	1 (2.0%)	0.631 ^4^
Chronic heart failure	11 (9.6%)	4 (6.2%)	7 (14.0%)	0.205 ^4^
Etiology of chronic kidney disease:				0.983 ^4^
Nephroangiosclerosis	18 (15.8%)	11 (17.2%)	7 (14.0%)	
Diabetic Nephropathy	31 (27.2%)	18 (28.1%)	13 (26.0%)	
Interstitial Nephropathy	9 (7.9%)	4 (6.3%)	5 (10.0%)	
Glomerulonephritis	15 (13.2%)	8 (12.5%)	7 (14.0%)	
Genetic diseases	7 (6.1%)	4 (6.3%)	3 (6.0%)	
Nephrotoxins	3 (2.6%)	1 (1.6%)	2 (4.0%)	
Others	13 (11.4%)	8 (12.5%)	5 (10.0%)	
Unknown	18 (15.8%)	10 (15.6%)	8 (16.0%)	
Laboratory data:				
Albumin (g/dL)	4.0 ± 0.3	4.1 ± 0.3	3.9 ± 0.3	0.010 ^1^
Prealbumin (mg/dL)	24.2 [19.9–28.8]	25.6 [22.3–30.1]	22.3 [18.8–26.7]	0.005 ^2^
Creatinine (mg/dL)	6.6 [5.6–8.2]	6.9 [5.7–8.8]	6.5 [5.6–7.5]	0.133 ^2^
IL-6 (pg/mL)	8.9 [5.9–16.4]	7.6 [4.8–11.4]	12.3 [7.1–19.0]	0.005 ^2^
CRP (mg/L)	4.7 [1.9–14.3]	3.3 [1.5–8.8]	8.5 [2.3–19.8]	0.011 ^2^
Sarcopenia Assessment Evaluations:				
SARC-F Survey				<0.001 ^3^
Normal (<4)	77 (67.0%)	53 (81.5%)	24 (48.0%)	
Abnormal (≥4)	38 (33.0%)	12 (18.5%)	26 (52.0%)	
Handgrip Dynamometer (kg)	19.8 [13.1–25.9]	23.0 [17–31.3]	14.5 [10.1–20.3]	<0.001 ^2^
Normal (≥27 kg Male; ≥16 kg Female)	35 (30.4%)	35 (53.8%)	0 (0.0%)	<0.001 ^3^
Abnormal (<27 kg Male; <16 kg Female)	80 (69.6%)	30 (46.2%)	50 (100.0%)	
BIS				
ASMI (kg/m^2^)	6.7 ± 1.4	7.5 ± 1.3	5.6 ± 0.8	<0.001 ^1^
Normal (≥7 kg Male; ≥5.5 kg Female)	56 (48.7%)	56 (86.2%)	0 (0.0%)	<0.001 ^3^
Abnormal (<7 kg Male; <5.5 kg Female)	59 (51.3%)	9 (13.8%)	50 (100.0%)	
TBW (L)	33.3 ± 7.3	36.9 ± 6.9	28.7 ± 4.7	<0.001 ^1^
BCM	16.9 [13.3–22.0]	20 [15.8–25.1]	14.4 [11.2–17.1]	<0.001 ^2^
PhA (degrees)	4.4 [3.8–5.5]	5.2 [4.2–6.3]	4.1 [3.7–4.5]	<0.001 ^2^
LTI (kg/m^2^)	12.3 ± 3.2	13.5 ± 3.4	10.7 ± 2.2	<0.001 ^1^
FTI (kg/m^2^)	14.4 [11.2–19.1]	16.9 [11.9–20.6]	12.8 [10.7–15.8]	0.006 ^2^
Ultrasound (US)				
CSA (cm^2^)	3.9 [3.1–5]	4.6 [3.6–5.7]	3.5 [2.8–4.2]	<0.001 ^2^
RFT (mm)	11.8 [9.7–14]	12.5 [10.8–16]	10.2 [8.5–12]	<0.001 ^2^

Data expressed as mean ± SD, median [p25–p75], or number (%). ^1^ T-Test Student, ^2^ Mann–Whitney U test; ^3^ Pearson’s Chi-squared test; ^4^ Fisher’s exact test. Abbreviations: BMI: Body mass index; MIS: Malnutrition Inflammation Score; BIS: Bioelectrical Impedance Spectroscopy; ASMI: Appendicular Skeletal Muscle Index; TBW: Total body water; BCM: Body Cell Mass; PhA: Phase Angle: LTI: Lean Tissue Index; FTI: Fat Tissue Index; CSA: Cross-Sectional Area; RFT: Rectus Femoris Muscle Thickness. Data expressed as mean ± SD, median [p25–p75], or number (%).

**Table 3:** Diagnostic accuracy of ultrasound and bioimpedance-derived muscle parameters for EWGSOP2-defined sarcopenia.

	AUC (IC 95%)	*p*-Value ^1^	Youden Cut-Off	Sensitivity (%) (IC 95%)	Specificity (%) (IC 95%)	PPV (%) (IC 95%)	NPV (%) (IC 95%)	LR+ (IC 95%)	LR− (IC 95%)
**Body Cell Mass (Kg)**	0.77 (0.68–0.85)	<0.001	17.65	80.0 (67.0–88.8)	61.5 (49.4–72.4)	61.5 (49.4–72.4)	80.0 (67.0–88.8)	2.1 (1.5–2.9)	0.3 (0.2–0.6)
Male	0.81 (0.72–0.91)	<0.001	18.85	78.1 (61.3–89.0)	75.6 (61.3–85.8)	69.4 (53.1–82.0)	82.9 (68.7–91.5)	3.2 (1.9–5.5)	0.3 (0.2–0.6)
Female	0.75 (0.58–0.91)	0.010	15.2	88.9 (67.2–96.9)	65.0 (43.3–81.9)	69.6 (49.1–84.4)	86.7 (62.1–96.3)	2.5 (1.4–4.7)	0.2 (0.04–0.7)
**Phase Angle**	0.75 (0.66–0.84)	<0.001	4.80	86.0 (73.8–93.1)	58.45(46.3–69.6)	61.4 (49.7–72.0)	84.4 (71.2–92.3)	2.1 (1.5–2.8)	0.2 (0.1–0.5)
Male	0.78 (0.68–0.88)	<0.001	4.47	71.9 (54.6–84.4)	73.3 (59.0–84.0)	65.7 (49.2–79.2)	78.6 (64.1–88.3)	2.7 (1.6–4.6)	0.4 (0.2–0.7)
Female	0.66 (0.47–0.84)	0.105	4.78	100 (82.4–100)	45.0 (25.8–65.8)	62.1(44.0–77.3)	100.0 (70.1–100)	1.8 (1.2–2.7)	0.00 (0.00–NaN)
**Rectus Femoris Muscle Thickness (mm)**	0.73 (0.63–0.82)	<0.001	11.85	74.0 (60.5–84.1)	66.2 (54.0–76.5)	62.7 (50.0–73.9)	76.8 (64.2–85.9)	2.2 (1.5–3.2)	0.4 (0.2–0.7)
Male	0.73 (0.62–0.85)	<0.001	11.85	65.6 (48.3–79.6)	73.3 (59.0–84.0)	63.6 (46.6–77.8)	75.0 (60.6–85.4)	2.5 (1.4–4.3)	0.5 (0.3–0.8)
Female	0.71 (0.54–0.88)	0.026	11.95	88.9 (67.2–96.9)	50.0 (29.9–70.1)	61.5 (42.5–77.6)	83.3 (55.2–95.3)	1.8 (1.1–2.8)	0.2 (0.1–0.9)
**Cross-Sectional Area (cm^2^)**	0.72 (0.63–0.81)	<0.001	4.44	84.0 (71.5–91.7)	53.9 (41.9–65.4)	58.3 (46.8–69.0)	81.4 (67.4–90.3)	1.8 (1.4–2.4)	0.3 (0.2–0.6)
Male	0.72 (0.61–0.84)	<0.001	4.44	75.0 (57.9–86.8)	62.2 (47.6–74.9)	58.5 (43.4–72.2)	77.8 (61.9–88.3)	2.0 (1.3–3.0)	0.4 (0.2–0.8)
Female	0.71 (0.55–0.88)	0.025	3.69	83.3 (60.8–94.2)	55.0 (34.2–74.2)	62.6 (42.7–78.8)	78.6 (52.4–92.4)	1.9 (1.1–3.1)	0.3 (0.1–0.9)

AUC: Area Under the Curve; PPV: Positive Predictive Value; NPV: Negative Predictive Value; LR+: Positive Likelihood Ratio; LR−: Negative Likelihood Ratio; BCM: Body Cell Mass. Mann–Whitney U Test.

## Data Availability

Data supporting the findings of this study are not publicly available due to patient privacy and institutional ethical restrictions, but are available from the corresponding author on reasonable request and subject to approval by the local ethics committee.

## Abbreviations

The following abbreviations are used in this manuscript:

ASMI	Appendicular Skeletal Muscle Index
BCM	Body Cell Mass
BIA	Bioelectrical Impedance Analysis
BIS	Bioimpedance Spectroscopy
BMI	Body Mass Index
CKD	Chronic Kidney Disease
CRP	C-reactive Protein
CSA	Cross-Sectional Area
CT	Computed Tomography
DXA	Dual-Energy X-ray Absorptiometry
EWGSOP2	European Working Group on Sarcopenia in Older People 2
FTI	Fat Tissue Index
HD	Hemodialysis
HG	Handgrip Strength
IL-6	Interleukin 6
MRI	Magnetic Resonance Imaging
PEW	Protein-Energy Wasting
PhA	Phase Angle
RFT	Rectus Femoris Muscle Thickness
ROC	Receiver Operating Characteristic
SARC-F	Strength, Assistance with walking, Rise from a chair, Climb stairs, and Falls questionnaire
TBW	Total Body Water
